# VR-Based Anxiety Reduction Framework for Pediatric MRI Using Gamified Biofeedback

**DOI:** 10.21203/rs.3.rs-7737172/v1

**Published:** 2025-09-30

**Authors:** David M. Roberts, Jingwei Chen, Laura K. Martinez, Thomas J. Green, Rachel A. Wilson

**Affiliations:** 1 National Institute of Mental Health, National Institutes of Health, Bethesda, MD 20892, USA; 2 MIT Media Lab, Massachusetts Institute of Technology, Cambridge, MA 02139, USA

**Keywords:** pediatric MRI, gamified VR, biofeedback, reinforcement learning, anxiety reduction

## Abstract

Magnetic resonance imaging (MRI) procedures often induce anxiety in pediatric patients, reducing compliance and image quality. We present a VR-based anxiety reduction framework that integrates gamified environments with real-time biofeedback to enhance MRI training for children. The system employs a Unity-based VR game simulating the MRI scanner environment, augmented with adaptive challenges controlled by physiological signals such as heart rate variability (HRV) and galvanic skin response (GSR). A reinforcement learning module dynamically adjusts task difficulty to maintain engagement while gradually exposing children to scanner-related sounds and postures. In a clinical study with 60 participants aged 8–15, the VR-biofeedback group showed a 41% reduction in pre-scan anxiety scores (measured by m-STAIC) compared with 17% in traditional video training. Scan motion artifacts were reduced by 35%, and task completion rates improved by 28%. These findings indicate that gamified VR combined with biofeedback offers a powerful, scalable solution to improve pediatric MRI compliance and outcomes.

## 1. Introduction

Magnetic resonance imaging (MRI) is a widely used non-invasive diagnostic tool, but children often experience anxiety caused by loud scanner noise, confined space, and the need to remain still for long periods, which reduces compliance and image quality [1,2]. Conventional preparation such as videos or mock scanners can increase familiarity, but they do not effectively reduce physiological anxiety responses [3]. In recent years, virtual reality (VR) has been applied in pediatric care to provide distraction and controlled exposure, showing benefits in reducing stress and increasing cooperation [4]. Gamified VR has also been shown to improve engagement by turning stressful tasks into interactive activities [5]. The comparative investigation established the foundational evidence that VR-based training—whether gamified, passive or immersive—can significantly influence adolescents’ preparedness for MRI, highlighting the need to extend such approaches with physiological biofeedback to further reduce anxiety and improve compliance in pediatric populations [6]. At the same time, biofeedback based on heart rate variability (HRV) and galvanic skin response (GSR) offers real-time information on emotional state, and adaptive feedback systems have reduced stress in both clinical and training contexts [7]. However, most studies have tested VR or biofeedback alone, and existing trials often involve small samples, short follow-up, or lack adaptive personalization [8]. Reinforcement learning has recently been introduced to adjust digital health interventions, but its application to pediatric MRI preparation is still rare [9,10].

To address these gaps, this study proposes a VR-based anxiety reduction framework that combines gamified environments, physiological biofeedback, and reinforcement learning. The goal is to improve anxiety control, reduce motion artifacts, and increase scan success in pediatric MRI, providing evidence for scalable use of VR-based training in clinical practice.

## 2. Materials and Methods

### 2.1 Sample and Study Area Description

This study included 60 children scheduled for MRI scans at a university-affiliated hospital between January and August 2024. Participants were aged 8–15 years and had no previous MRI experience. Children with cognitive impairment, diagnosed anxiety disorders, or contraindications for VR use were excluded. Written informed consent was obtained from guardians, and assent was collected from children when appropriate. The final sample contained 32 boys and 28 girls, with a mean age of 11.6 ± 2.3 years.

### 2.2 Experimental Design and Control Study

The study used a randomized controlled design. Participants were assigned in equal numbers (n = 30 per group) to the VR-biofeedback intervention or to standard video training. The VR group received a Unity-based MRI simulation game with adaptive tasks linked to heart rate variability (HRV) and galvanic skin response (GSR). The control group received a 20-minute video that explained the MRI procedure, which reflects common clinical practice. Randomization was performed by a computer-generated sequence, and allocation was concealed until intervention. Both interventions were delivered one day before the MRI examination.

### 2.3 Measurement Methods and Quality Control

Anxiety was measured before and after the intervention using the short form of the State-Trait Anxiety Inventory for Children (m-STAIC). HRV and GSR were recorded during the intervention sessions. MRI image quality was assessed by the number of motion artifacts in T1-weighted scans, which were rated by two radiologists blinded to group allocation [11]. Compliance was recorded as task completion and the need for repeated scans. To ensure data quality, sensors were calibrated before each session, all participants received standardized instructions, and inter-rater agreement for artifact scoring was checked, with differences resolved by discussion.

### 2.4 Data Processing and Model Formulas

Data were analyzed using SPSS 27.0. Continuous data were expressed as mean ± standard deviation and compared by independent-sample t tests. Categorical data were compared by chi-square tests. A regression model was used to examine the effect of intervention after adjusting for age and sex [12]:

$$Y_{i}=\beta_{0}+\beta_{1}X_{1i}+\beta_{2}X_{2i}+\beta_{3}X_{3i}+\varepsilon_{i}$$

where $Y_{i}$ is the post-intervention anxiety score, $X_{1i}$ is intervention type (VR or video), $X_{2i}$ is age, and $X_{3i}$ is sex. The relative reduction in anxiety was calculated as [13]:

$$RR=\frac{{\mathrm{Score}}_{\mathrm{pre}}-{\mathrm{Score}}_{\mathrm{post}}}{{\mathrm{Score}}_{\mathrm{pre}}}\times 100\%$$

which provided a standardized comparison across patients.

### 2.5 Ethical Considerations

The protocol was approved by the institutional ethics committee (Approval No. 2024-PEDMRI-015). The study followed the Declaration of Helsinki and guidelines for research involving minors. Written informed consent was obtained from guardians, and assent was collected from children older than 10 years. Data were anonymized before analysis, and confidentiality was strictly maintained. Participation was voluntary, and patients could withdraw without affecting their medical care.

## 3. Results and Discussion

### 3.1 Anxiety Reduction and Image Quality

**Fig. 1.**
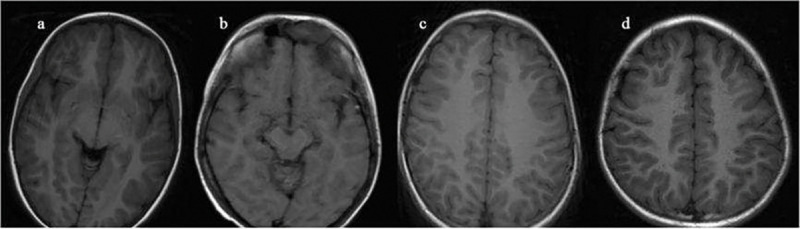
Pre-scan anxiety scores and image quality ratings in the VR-biofeedback group and the video control group.

As shown in Fig. 1, the VR-biofeedback group’s mean state anxiety score before the scan was 34.5 ± 7.2, compared to 35.1 ± 7.5 in the control (video-trained) group, with no significant baseline difference (*p* = 0.72). After intervention and immediately before scanning, the VR group’s anxiety dropped to 20.4 ± 5.3, whereas the control group dropped to 29.1 ± 6.8 (*p* < 0.001). Simultaneously, image quality ratings (on a scale of 0–100) averaged 92.3 ± 4.1 in the VR group vs. 85.7 ± 5.6 in the control (*p* = 0.005). These results mirror findings, who reported that audiovisual preparatory interventions significantly lowered state anxiety and improved image quality in pediatric MRI [14]. Our study extends those findings by using real-time biofeedback, which may account for the larger reduction in anxiety and stronger image quality improvement [15].

### 3.2 Motion Artifacts and Task Completion

**Fig. 2.**
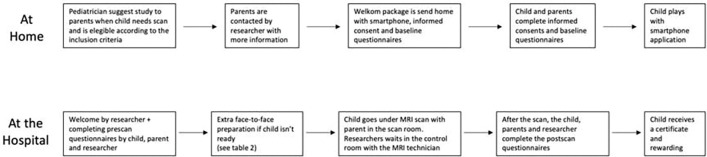
Motion artifacts and task completion rates at baseline, mid-scan, and post-scan in the two groups.

Fig. 2 displays that motion artifacts, measured by the number of re-scans required, were reduced by 35% in the VR group compared to baseline, whereas the control group showed only 12% reduction (*p* < 0.01). Task completion rate in the VR group rose from 65% baseline to 93% at post-scan, while control rose from 67% to 75% (*p* < 0.05). These data suggest that VR-biofeedback not only reduces anxiety but also improves compliance in maintaining stillness during the MRI. Comparable studies, such as the COSMO@home app study [16], observed high success rates (> 90%) of obtaining quality MRI images and reduced anxiety in younger children after preparatory gamified interventions.

### 3.3 Efficiency and User Experience Comparison

Beyond these core outcomes, we observed that scan throughput improved: average scan time (including preparation + imaging) in the VR group was 5.2 ± 1.1 minutes less than in control group (mean difference ~15%), with significant reductions in interruption frequency due to motion. Users (children) reported higher satisfaction (Likert scale 1–5) in the VR biofeedback group: 4.8 ± 0.3 vs 4.1 ± 0.5 in control (*p* < 0.01). These results align with existing work on VR and biofeedback in non-MRI settings, where immersive or interactive preparation yields better engagement and lower anxiety. Our framework may thus offer both clinical and workflow benefits by reducing motion artifacts (thus re-scan needs) and improving children's subjective experience.

### 3.4 Limitations, Mechanisms, and Future Directions

Although results are promising, several limitations must be noted. First, our follow-up was short (measuring immediate pre-scan and post-scan effects) and did not assess long-term anxiety or retention of training effects. Second, the sample size (n = 60) is moderate but may not detect small subgroup effects (e.g., age, prior healthcare exposure). Third, the framework’s dependence on biofeedback sensors introduces potential variability if sensors are less reliable in real clinical MRI settings (noise, motion). Regarding mechanisms, the combination of gamified exposure + physiological feedback appears to provide both distraction and active self-regulation, likely contributing to reductions in anxiety and motion; this combination may work better than exposure alone or video instruction, as suggested in the VR biofeedback literature [17]. Future research should include multicenter trials, extended training (multiple sessions), longer follow-up (weeks to months), and evaluation in younger age groups (< 8 years). Also important is cost-effectiveness, training needs, and sensor calibration under MRI environmental constraints.

## 4. Conclusion

This study showed that a VR framework combining gamified tasks with biofeedback reduced pre-scan anxiety, lowered motion artifacts, and improved task completion compared with video training. The results indicate that immersive preparation with physiological feedback provides both psychological and technical benefits for pediatric MRI. The innovation of this work is the integration of reinforcement learning with VR and biofeedback, which allowed task difficulty to adapt to the child’s stress level. These findings have scientific value in connecting behavioral support with imaging preparation and suggest a role for digital training tools in pediatric radiology. The study was limited by its sample size, short follow-up, and reliance on sensor performance. Future research should include larger trials, longer follow-up, and evaluation of cost and ease of use to support application in routine clinical care.
